# Relapsing Polychondritis: A Rare Case Report

**DOI:** 10.7759/cureus.40172

**Published:** 2023-06-09

**Authors:** Ilakyaa Rajakumar, Kavyaashree Karthikeyan, Pugazhvanan C R, Aamina Hussain, Krishnaswamy Madhavan

**Affiliations:** 1 General Medicine, SRM Medical College Hospital and Research Centre, Chengalpattu, IND; 2 General Medicine, Apollo Hospitals, Chennai, IND; 3 Community Medicine, SRM Medical College Hospital and Research Centre, Chengalpattu, IND

**Keywords:** otomastoiditis, laryngotracheobronchomalacia, relapsing polychondritis, auriculitis, saddle nose deformity

## Abstract

Relapsing polychondritis is an uncommon disorder of unknown cause characterized by inflammation of cartilage, predominantly affecting the ear, nose, and laryngotracheobronchial tree. The case under discussion is a 50-year-old female with a classical presentation of relapsing polychondritis with saddle nose deformity, bilateral auriculitis, and laryngotracheobronchomalacia with joint involvement.

## Introduction

Relapsing polychondritis is a rare connective tissue disorder. It is a severe, episodic, and progressive inflammatory condition involving cartilaginous structures and proteoglycan-rich tissues such as the nose, ears, larynx, trachea, blood vessels, heart, cornea, sclera, kidney, and joints [1-3]. The first case of relapsing polychondritis was reported in 1923 with the term “polychondropatia,” and its current definition was later given by Pearson et al. in 1960 [4-5]. The estimated incidence of this rare multisystem disease is around 3.5 cases per million per year [6]. The peak age of onset of relapsing polychondritis is between the ages of 40 and 50, but it could affect children and the elderly [7-9]. A study reported a female preponderance of 3:1 [10]. A higher frequency of HLA-DR4 has been found in patients with relapsing polychondritis than in healthy individuals [11]. Non-rheumatic disorders have been associated with relapsing polychondritis. Relapsing polychondritis is considered as a complex disorder as it targets the cartilaginous structure where both cell-mediated and humoral immunity plays an important role in its pathogenesis. The immune response to type 2 collagen and matrilin 1 is believed to play an important role in the pathogenesis [12]. Although less than half of patients have antibodies to type 2 collagen in sera, these are non-specific.

## Case presentation

A 50-year-old female presented with complaints of Grade 3 breathlessness according to the modified Medical Research Council (mMRC) for 3 days, wheezing, coughing, and mucoid scanty expectoration for 6 months. She is a known case of type 2 diabetes, systemic hypertension, bronchial asthma, and depression and was on regular medication.

**Figure 1:**
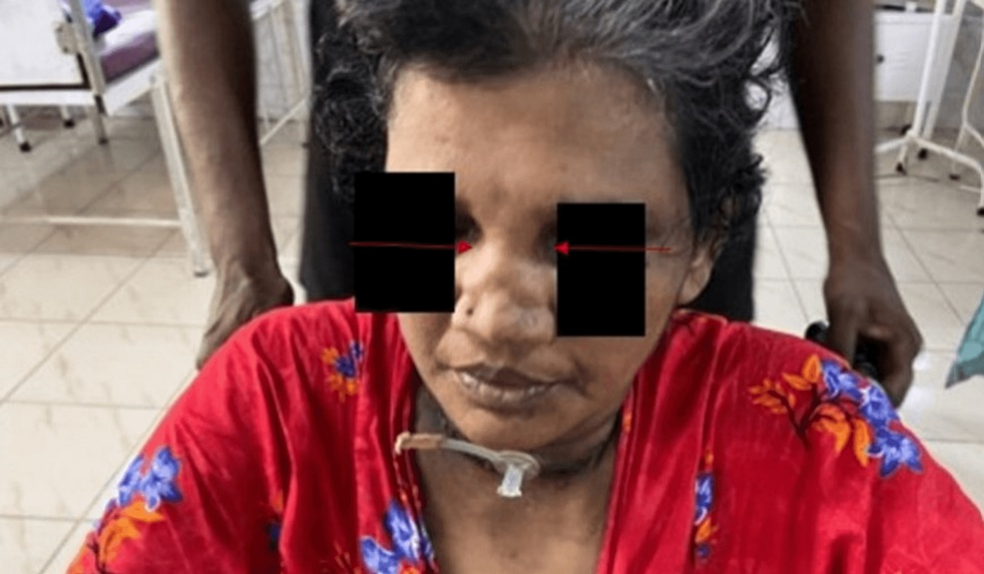
Saddle nose deformity.

On examination, the patient was found to have tachypnea, urticaria, and with saddle nose deformity. Figure 1 below shows the patient with saddle nose deformity. Respiratory auscultation revealed bilateral rhonchi and bilateral crepts. The investigation reports are listed in Table 1.

 

**Table 1:** Investigation I. LV, left ventricular; ABG, arterial blood gas; ECG, electrocardiogram; ANA, antinuclear antibody; ESR, erythrocyte sedimentation rate

Investigation	Results
ESR	23 mm/h
C-reactive protein	17.7 mg/dL
ANA	1+ nucleolar pattern
Pulmonary function test	Obstructive pattern
Ophthalmic examination	No features of ocular inflammation
ECG	Normal sinus rhythm
Echocardiogram	Normal LV function, normal chambers, no valvular abnormality
ABG	Respiratory acidosis

As the patient was desaturated on room air, arterial blood gas (ABG) revealed respiratory acidosis. The patient was started on noninvasive ventilation with FiO2 at 50%, and her saturation improved to 100%. The patient was treated with nebulized bronchodilators and steroids. Eventually, the patient improved symptomatically and was discharged.

One month later:

The patient presented in a drowsy state with complaints of breathlessness.

On examination:

The patient had biphasic stridor, and auscultation revealed bilateral, extensive wheeze. The investigation reports are shown in Table 2.

**Table 2:** Investigation II. ABG, arterial blood gas

Investigation	Results
ABG	Type 2 respiratory failure
Fiber optic bronchoscopy	Bilateral abducted vocal cords with laryngeal edema with subglottic stenosis
CT neck	Asymmetric circumferential wall thickening of the subglottic larynx with focal loss of fat plane with esophagus posteriorly causing subglottic stenosis
Rigid bronchoscopy-guided subglottic biopsy	Fragments of pseudo-stratified ciliated columnar epithelium and stratified squamous epithelium with underlying edematous stroma showing lymphoplasmacytic infiltrates along with few congested vessels

Therefore, the patient was taken up for an emergency tracheostomy.

Two months later:

The patient was brought to the emergency room in a drowsy state, responding only to painful stimuli and complaining of breathing difficulty.

**Figure 2:**
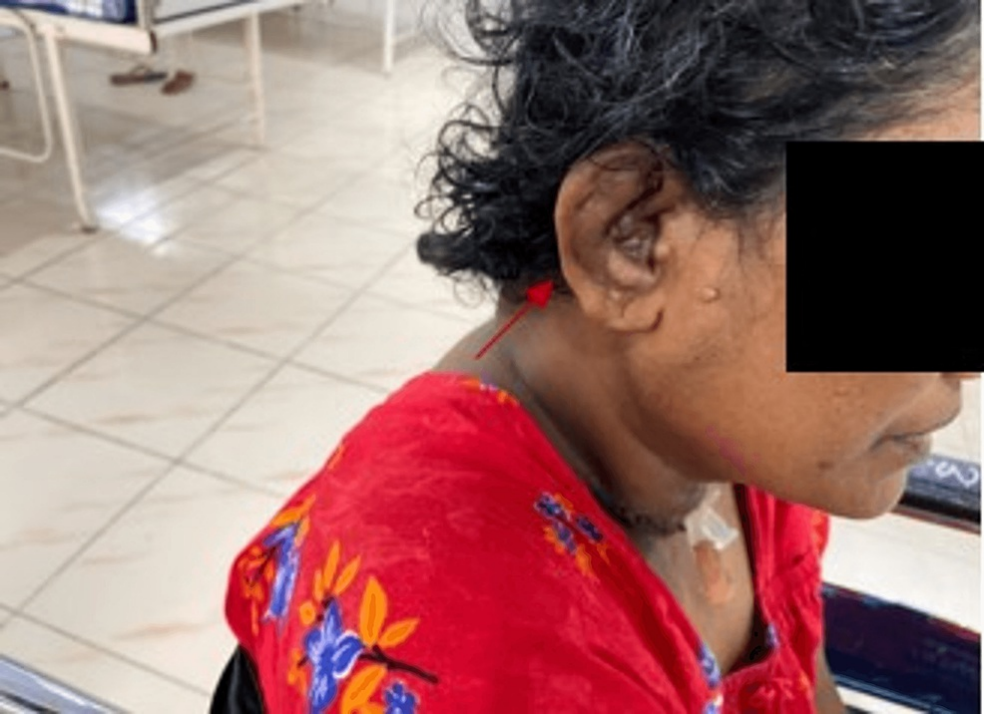
Auriculitis.

On examination:

The patient had violaceous discoloration and swelling of bilateral ear lobes (Figure 2), sparing the pinna; auscultation revealed bronchial breathing in the right upper lobe.

The patient was diagnosed with pneumonia and was treated with appropriate antibiotics. During the same admission, the patient developed swelling and tenderness over the proximal interphalyngeal (PIP) joint, the distal interphalyngeal (DIP) joint of the right hand, and the left ankle. The investigation reports are shown in Table 3.

**Table 3:** Investigation III. ESR, erythrocyte sedimentation rate; CRP, C-reactive protein; RF, rheumatoid factor

Investigation	Results
CT paranasal sinus	Chronic right otomastoiditis
ESR	25 mm/h
CRP	12 mg/dL
RF	Negative

Diagnosis:

Given the patient’s saddle nose deformity, bilateral auriculitis, laryngotracheobronchomalacia, and asymmetric arthritis, the patient was suspected to have relapsing polychondritis as per the diagnostic criteria as shown in Table 4.

**Table 4:** Diagnostic criteria.

Reference	Symptoms	Diagnostic requirements
McAdam et al. [13]	Recurrent chondritis in both auricles, non-erosive inflammatory arthritis, chondritis of nasal cartilage, inflammation of ocular structures, chondritis of the laryngeal and/or tracheal cartilage neurosensory hearing loss	Patient with ≥ 3 symptoms
Damiani et al. [14]	1. Patients meeting ≥3 standards in McAdam’s criteria 2. >1 standard in McAdam’s criteria, plus positive biopsy 3. Lesions involving ≥2 anatomical sites of cartilage inflammation, responded to glucocorticoids or dapsone.	Patient meeting any one of the criteria

**Figure 3:**
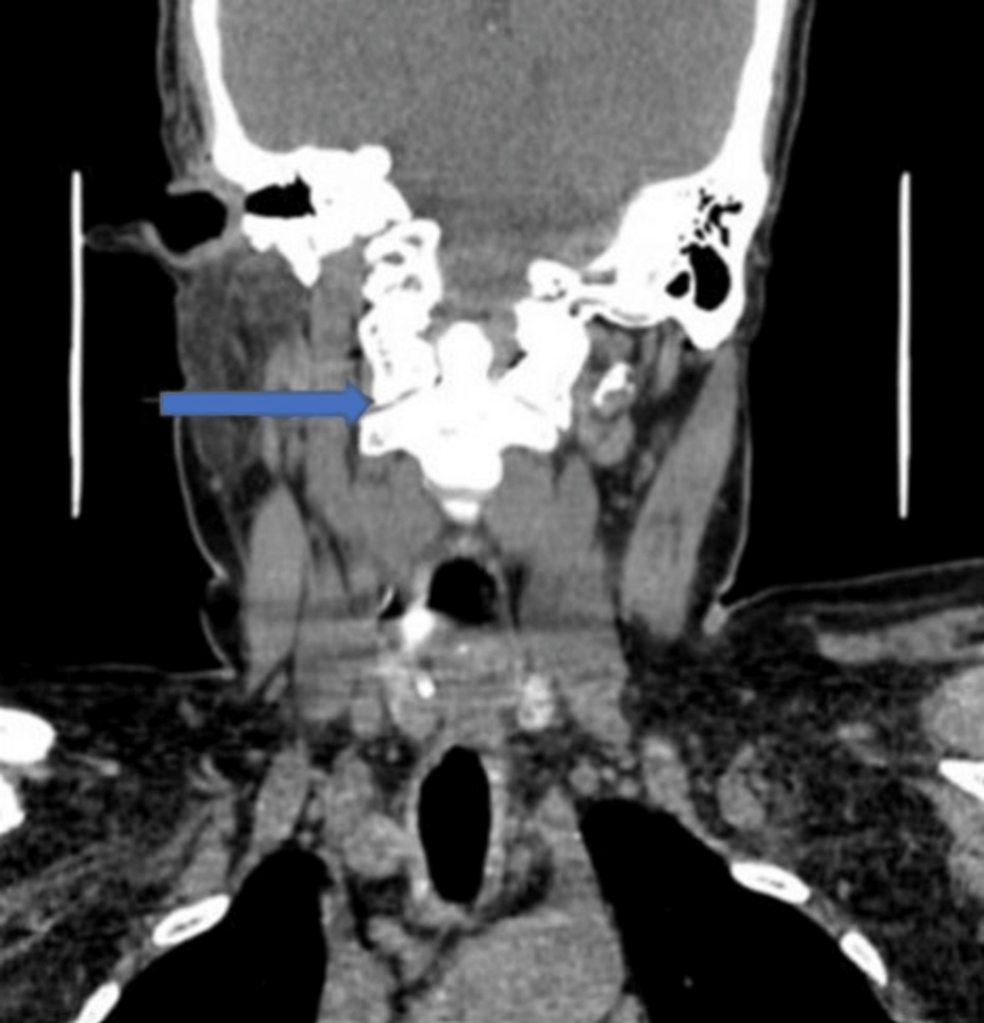
CT neck showing subglottic stenosis.

**Figure 4:**
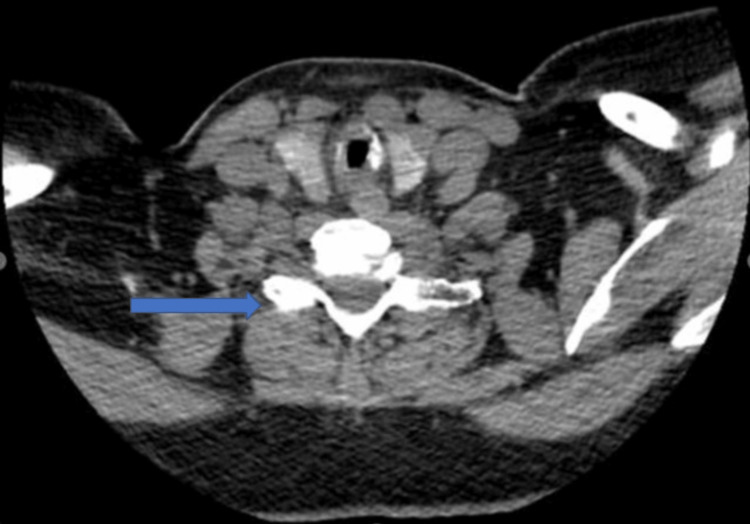
CT neck showing subglottic stenosis.

A CT neck showing asymmetric circumferential wall thickening of the subglottic larynx with focal loss of the fat plane with the esophagus posteriorly causing subglottic stenosis is shown in Figures 3-4.

**Figure 5:**
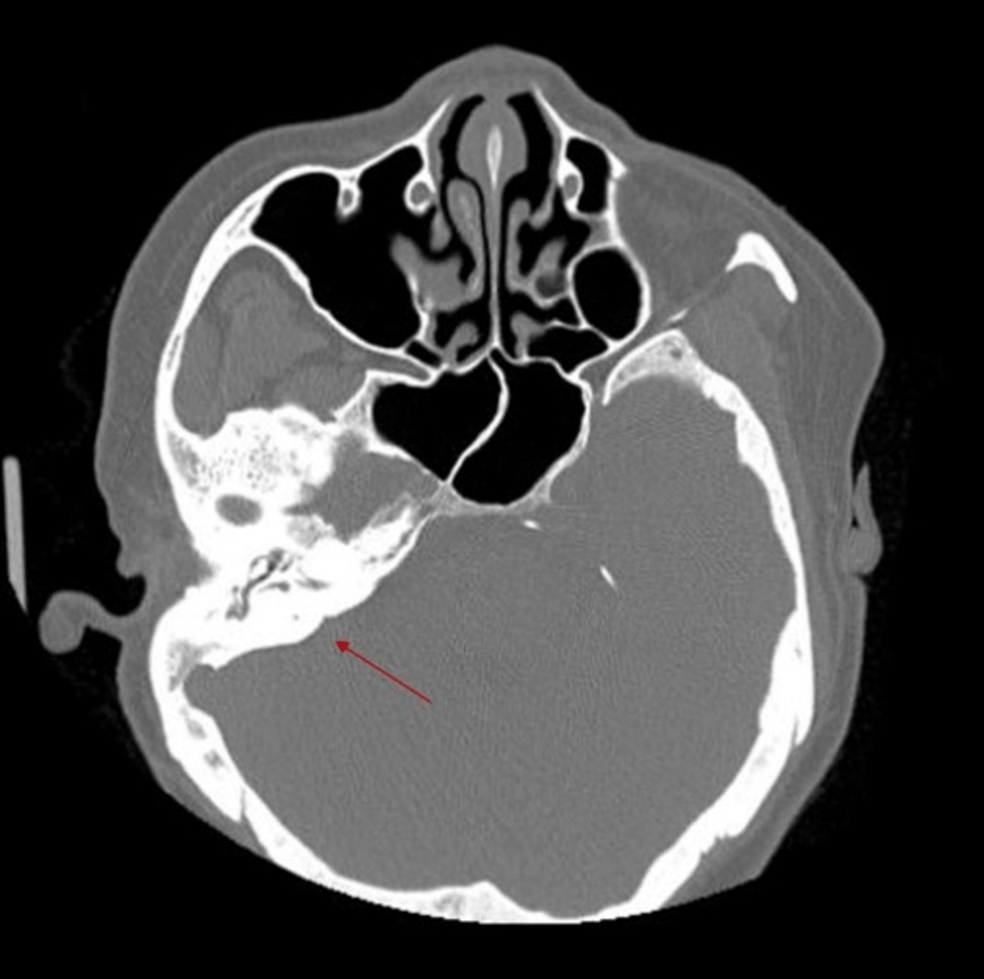
CT paranasal sinus showing right otomastoiditis.

A CT paranasal sinus showing chronic right otomastoiditis is shown in Figure 5.

## Discussion

Relapsing polychondritis is a rare disease with abrupt onset. It is a progressive autoimmune disease with multisystem involvement and fatal complications. The symptoms vary in the initial stages of the disease according to the organs affected [15]. The most common site involved is the auricular cartilage, and the earlobe is spared. However, due to repeated attacks, cauliflower ear may develop along with cartilage damage. Even though the nasal cartilage is a less involved location, it is very painful; along with obstruction, there is epistaxis, discharge, and scabbing as mentioned in many articles [16]. As seen in the current case, saddle nose deformities can occur after repeated attacks. Bilateral auriculitis occurred after saddle nose deformity in this patient. In relapsing polychondritis, joint involvement is common. Usually, it is asymmetric, non-erosive, and seronegative oligo/polyarthritis [17]. The studied patient developed non-erosive, seronegative polyarthritis. In some cases, due to atypical clinical symptoms, relapsing polychondritis with laryngotracheal involvement is difficult to diagnose as it is easily confused with other diseases such as bronchitis, Adam’s apple tuberculosis, and asthma. Usually, diagnosis is based on the recognition of typical clinical features. A biopsy of the involved cartilage from the ear, nose, or respiratory tract can confirm the diagnosis, but it is only necessary when symptoms are not typical. The diagnostic criteria proposed by McAdam [13] in 1976 were modified by Damiani and Levine in 1979 [14]. These criteria have been mentioned as standard criteria by many other authors as well [18-20]. Even though this is the standard criteria, certain authors have developed alternative criteria because some patients do not meet McAdam’s criteria.

## Conclusions

Based on McAdam’s criteria for diagnosis, the patient under study was diagnosed with relapsing polychondritis as she exhibited four typical clinical features (saddle nose deformity, bilateral auriculitis, laryngotracheobronchomalacia, non-erosive seronegative polyarthritis, and a positive biopsy). Therefore, the patient was started on oral glucocorticoids, including methotrexate. The patient’s arthritis was resolved and breathlessness improved. Steroids were gradually tapered and stopped as the patient’s symptoms improved. The patient is planned for tracheal reconstruction. In conclusion, the patient is a classic case of relapsing polychondritis as the clinical features satisfy the criteria.
